# Low-Cost Sensors and Biological Signals

**DOI:** 10.3390/s21041482

**Published:** 2021-02-20

**Authors:** Frédéric Dierick, Fabien Buisseret, Stéphanie Eggermont

**Affiliations:** 1 Laboratoire d’Analyse du Mouvement et de la Posture, Centre National de Rééducation Fonctionnelle et de Réadaptation-Rehazenter, 2674 Luxembourg, Luxembourg; 2 Faculty of Motor Sciences, Université Catholique de Louvain, 1348 Louvain-la-Neuve, Belgium; 3 CeREF-Technique, Chaussée de Binche 159, 7000 Mons, Belgium; buisseretf@helha.be (F.B.); stephanie.eggermont@cerisic.be (S.E.); 4 Service de Physique Nucléaire et Subnucléaire, UMONS, Research Institute for Complex Systems, Place du Parc 20, 7000 Mons, Belgium

Low-cost sensors, i.e., sensors typically cheaper than USD 100, are currently available, allowing the measurement of a wide range of physiological signals. These signals contain valuable information that can be used to increase the understanding of any physiological function of clinical interest. Hence, low-cost sensors are expected to play a key role in the future of clinical practice and medical diagnosis. In particular, they may facilitate the collection of big data and allow broader diffusion of evidence-based medicine, which is essential to improving medical practice. Low-cost sensors may also be of interest in virtual or augmented reality applications, including rehabilitation. Their use is associated with several challenges: First, sensors should be accurate enough to unambiguously compute relevant indicators from biosignals, in particular in patients with medical conditions. Second, the designed sensors should be as non-intrusive and ready-to-use as possible with fast calibration procedures. Third, they require user-friendly and cross-platform interfaces that provide secure data storage and easy data analysis and visualization. We invited authors to submit their latest results in the field, either research articles or reviews; 12 papers were accepted for publication in this Special Issue of *Sensors,* entitled “Low-Cost Sensors and Biological Signals.” They are summarized in the next paragraphs.

Low-cost sensors allow for the full monitoring of human motion. In particular, inertial measurement units (IMUs) and magnetic angular rate and gravity sensors (MARGs) are compact devices able to measure the 3D acceleration and angular speed of a given anatomical landmark with an accuracy comparable to gold-standard material only available in research centers [1]. As shown in this work through the study of a clinical test assessing neck mobility, the precision reached is sufficiently high for daily use in clinical practice. More generally, physiotherapy is a field that can benefit from such motion sensors. Cappelle et al. [2] present a low-complexity wireless motion sensor based on IMUs designed to be physiotherapist-friendly. The small size and low weight as well as the wireless data transmission are needed to reduce the impact on patient motion and to allow for easy positioning on a patient’s body.

Regarding daily use, calibration has to be as fast as possible compared to the typical time a clinician spends with a patient. Accurate calibration will allow the computation of angular position from acceleration and angular velocity. Angular amplitudes are one of the most commonly used indices to assess joint mobility. Calibration procedures are presented [3] for IMUs, leading to an accuracy of less than 3.4° on lower limb amplitude measurements. These results are coherent with those of Hage et al. [1], although Hage et al. focused on the neck rather than lower limbs. In real-life situations, some perturbations cannot be avoided, which jeopardize calibration efforts, e.g., magnetic disturbances for MARGs. It may be necessary to add extra information to compensate for the perturbations. An example is given in Wöhle and Gebhard [4], who show that eye-tracking data can be used to improve the accuracy on MARG head-orientation measurements.

Once human motion is measured, it can be used as an input signal to interact with a virtual environment or with more classical videogames. An example is provided in Foreman and Engsberg [5], who show that Microsoft Kinect^®^ is a reliable tool for assessing trunk motion. The coupling between low-cost motion sensors and serious videogames opens the possibility to innovative methods in rehabilitation. A review [6] shows that the use of videogames and motion-capture systems in rehabilitation contributes to the recovery of the patient, mostly in post-stroke rehabilitation. Sensors may be relevant not only in rehabilitation but also in helping patients to improve their motor abilities and to recover autonomy. Krasovsky et al. [7] focus on adults and children with motor impairments such as stroke or cerebral palsy. They show that a spoon instrumented with an IMU allows for a clinically feasible assessment of self-feeding.

Kinematics is obviously not the only method to assess physical activity. Two other types of biosignals are discussed [8,9]. In Tahir et al. [8], a systematic design and characterization procedure for different pressure sensors is proposed for building low-cost smart insoles for detecting vertical ground reaction force in gait analysis. In Wójcikowski and Pankiewicz [9], a new algorithm for the measurement of the human heart rate using photoplethysmography is presented. The algorithm is less demanding in computing power than many others, which is an important advantage regarding the autonomy of wearable devices.

Low-cost sensors may not only be useful in characterizing an individual’s state: they can also offer ways to classify individuals in different groups. Gabis et al. [10] show that accelerometers provide enough information to discriminate between typically developed children and children with autism spectrum disorder via a simple motor task (star jump). Li et al. [11] report that the kinematic patterns measured by IMUs are significantly different between the Baduanjin teacher, senior students, and junior students: changes in kinematics are, in this case, related to one participant’s experience.

A direction in which low-cost sensors may be applied is in affective technologies: biosignals measured by sensors (temperature, skin humidity, etc.) reflect the emotional state of an individual. Such signals may be communicated to a wearable device worn by another person to enhance the methods of communicating with each other [12].

## Data Availability

Not applicable.
